# Fantastic perspectives and where to find them: involving patients and citizens in digital health research

**DOI:** 10.1186/s40900-022-00374-6

**Published:** 2022-08-02

**Authors:** Esli Osmanlliu, Jesseca Paquette, Annie-Danielle Grenier, Paul Lewis, Marie-Eve Bouthillier, Sylvain Bédard, Marie-Pascale Pomey

**Affiliations:** 1 Research Institute of the McGill University Health Centre, Montreal, QC H4A 3J1 Canada; 2 Research Centre of the University of Montreal Hospital Centre, Montreal, QC H2X 0A9 Canada; 3 DIGICIT Advisory Committee, Research Centre of the University of Montreal Hospital Centre, Montreal, QC H2X 0A9 Canada; 4 Office of Clinical Ethics, Faculty of Medicine, University of Montreal, Montreal, QC H3C 3J7 Canada; 5 Centre of Excellence for Partnership with Patients and the Public (CEPPP), Montreal, QC H2X 0A9 Canada; 6 Department of Health Policy, Management and Evaluation, School of Public Health, University of Montreal, Montreal, QC H3N 1X9 Canada

**Keywords:** Patient and public involvement, Patients, Citizens, Co-construction, Partnership, Digital Health Research, Advisory Committee, App, Exposure notification, COVID-19

## Abstract

**Background:**

Digital contact tracing and exposure notification apps have quickly emerged as a potential solution to achieve timely and effective contact tracing for the SARS-CoV-2 virus. Nonetheless, their actual uptake remains limited. Citizens, including patients, are rarely consulted and included in the design and implementation process. Their contribution supports the acceptability of such apps, by providing upstream evidence on incentives and potential barriers that are most relevant to users. The DIGICIT (DIGITal CITizenship) project relied on patient and citizen partnership in research to better integrate public perspectives on these apps. In this paper, we present the co-construction process that led to the survey instrument used in the DIGICIT project and the interpretation of its results. This approach promotes public participation in research on contact tracing and exposure notification apps, as well as related digital health applications.

**Objectives:**

This article has three objectives: (1) describe the methodological process to co-construct a questionnaire and interpret the survey results with patients and citizens, (2) assess their experiences regarding this methodology, and (3) propose best practices for their involvement in digital health research.

**Methods:**

The DIGICIT project was developed in four steps**: (**1) creation of the advisory committee composed of patients and citizens, (2) co-construction of a questionnaire, (3) interpretation of survey results, and (4) assessment of the experience of committee participants.

**Results:**

Of the 25 applications received for participation in the advisory committee, we selected 12 people based on pre-established diversity criteria. Participants initially generated 84 survey questions in the first co-construction meeting, and eventually selected 36 in the final version. Participants made more than 20 recommendations when interpreting survey results and suggested carrying out focus groups with marginalized populations to increase representativity. They appreciated their inclusion early in the research process, being listened to and respected, the collective intelligence, and the method used for integrating their suggestions. They suggested that the study objectives and roles be better defined, that more time in the brainstorming sessions be allowed, and that discussion outside of meetings be encouraged.

**Conclusion:**

Having patients and citizens actively participating in this research constitutes the main methodological strength. They enriched the study from start to finish, and recommended the addition of focus groups to seek the perspective of marginalized groups that are typically under-represented from digital health research. Clear communication of the project objectives, good organization in meetings, and continuous evaluation from participants allow best practices to be achieved for patients' and citizens' involvement in digital health research. Co-construction in research generates critical study design ideas through collective intelligence. This methodology can be used in various clinical contexts and different healthcare settings.

**Supplementary Information:**

The online version contains supplementary material available at 10.1186/s40900-022-00374-6.

## Plain English summary

COVID Alert is a mobile application (app) that was developed created to help limit the spread of COVID-19 in Canada. Although promising, these apps have not been widely used by the population, in part due to limited citizen engagement in their design. The DIGICIT project (DIGITal CITizenship) was carried out in partnership with citizens, including patients, to gather public perspective in Quebec, Canada about the COVID Alert app. The purpose of this article is to describe our method of constructing a survey questionnaire with patients and citizens. We have created an advisory committee of 12 participants. Along with the research team, they created a 36-question survey. They also suggested doing focus groups to add data to the survey by reaching marginalized groups that are traditionally excluded from digital health research. We also wanted to know the experience of the participants being included in this study. We conducted interviews and did a small survey with them. They appreciated being included from the beginning of the research, being listened to, and being respected. They appreciated the creativity of the group and the brainstorming sessions. However, they would have liked the tasks to be clearer from the start. They also would have preferred to have more time in the brainstorming sessions to create the survey questions, and to have discussions outside of the meetings. The inclusion of patients and citizens is the main strength of this project. To improve their integration in health research, there needs to be good communication of project objectives. Also, meetings must be well organized, and participants must be able to evaluate their experience.

**Supplementary Information:**

The online version contains supplementary material available at 10.1186/s40900-022-00374-6.

## Introduction

### Digital contact tracing and exposure notification apps

Early in the pandemic, digital contact tracing and exposure notification apps generated substantial enthusiasm due to their potential for reducing transmission rates and enabling targeted lockdown strategies. However, although surveys demonstrate public interest in using such apps [1–8], their actual uptake remains limited [9]. The DIGICIT (DIGITal CITizenship) research project's global objectives included enriching the public debate through collaborative research, assessing citizen perspectives on contact tracing and exposure notification technologies in a public health emergency, and facilitating collaborations that promote responsible and ethical digital health innovation. This was achieved by having a patient as a principal investigator (S.B.), and by partnering with patients and citizens throughout the research project.

### Partnership with the public

In light of the many open questions surrounding the viability of app-based contact tracing, it is important to know the conditions for a socially acceptable use of such tools. Therefore, to help improve uptake, it could be highly beneficial to include a partnership with citizens, developers, researchers and decision-makers in the co-design of the application to ensure that the users’ specific needs are considered [10]. Indeed, including patients and citizens in research creates constructive exchanges with different kinds of knowledge, resources, competencies, and ideas, and also helps foster more efficient and effective solutions through collaborative efforts [11].

The process of research being conducted with or by members of the public and patients, as opposed to conducting research for or about them is the definition of Patient and Public Involvement (PPI). Its purpose is to "improve the quality, relevance, and impact of health research while improving transparency of the process and accountability to the wider community of researchers themselves" [12]. The literature indicates that six important actions are necessary in order to achieve a positive impact of PPI in research [13]: (1) the researchers and lay representatives having a shared understanding of the moral and methodological purpose of PPI, (2) a key individual coordinating PPI, (3) lay representatives having a strong connection with the target study population, (4) the whole research team being positive about PPI input and fully engaged with it, (5) efforts to develop relationships established and maintained over time and (6) PPI is evaluated in a proactive and systematic approach.

However, not all kinds of involvement are equal. The Montreal model [14] proposes four distinct levels along a patient/public engagement continuum, in ascending order of engagement: (1) information, (2) consultation, (3) collaboration, and (4) partnership. Partnership-focused frameworks put emphasis on measures taken to support partnership and assure transparency [15]. When in partnership, the patient is involved throughout the process and their lived experiences are taken into account. Their words are considered in the same manner as that of the other team members, be it researchers or research collaborators. Their involvement can, for example, take the form of their active participation in the development of the research protocol, the development of the methodology used, the analysis of the results, and the dissemination of research results. In addition, they can be involved in the identification of themes and recommendations for research. In digital health, patients and citizens can be consulted to gather their views or preferences about a health technology based on their life experience or their specific experience with illness [16]. In addition, researchers must be open-minded, able to work as a team and share information with all team members, including the patients. These perspectives could add key aspects to health technology assessment that might otherwise be overlooked [16]. Complementary forms of engagement can promote diversity in PPI, as the time and resources necessary for more engaged levels may be prohibitive for marginalized groups [17].

Despite its importance, PPI in digital health research remains limited. Given this gap in implementation, the academic community may benefit from published experiences with PPI-promoting methodologies. Thus, this article has three objectives: (1) to describe the methodological process of questionnaire co-construction and the interpretation of the survey results with patients and citizens, (2) to assess their experiences in regard to this methodology, and (3) to propose best practices for their involvement in digital health research.

## Methods and findings

The DIGICIT project was developed in four steps: (1) creation of the advisory committee composed of patients and citizens, (2) co-construction of a questionnaire on the COVID Alert app, (3) interpretation of the survey results, and (4) assessment of the participants’ experience. We report the results in accordance with the Guidance for Reporting Involvement of Patients and the Public Long Form (GRIPP2- LF; Additional file 1) [18] to guarantee the quality and reporting of patient and citizen involvement. This research project received ethics approval from the Research Ethics Committee (CER #20.276) of the Research Center of the University of Montreal Hospital Centre (CRCHUM).

### Step 1: Creation of the advisory committee (December 2020 to January 2021)

#### Methods

A call for applications (see Multimedia Appendix 1) to recruit between 10 and 12 people was posted at: (1) the Institut du Nouveau Monde (INM [19]), recognized for its expertise in the field of citizen involvement, (2) the National Institute of Excellence in Health and Social Services (INESSS [20]), a health technology assessment agency recognized for its patients and citizen implication, (3) the Centre of Excellence on Partnership with Patients and the Public (CEPPP [21]), a think tank that developed the best practices on PPI, and (4) the Québec community of practice on patient partnership [22], which aims to share best practices on PPI in the healthcare system.

To include a full range of viewpoints on what constitutes the socially acceptable and sustainable use of digital contact tracing and exposure notification apps in a public health emergency, five criteria for representativity and diversity were established to guide the composition of the advisory committee: (1) gender parity, (2) regional, generational and cultural diversity, (3) the state of health and the experience of living with a disease, (4) the sensitivity to the vulnerabilities that certain population groups may experience related to the use of contact notification technologies, and (5) the general attitudes towards contact notification technologies. The eligibility criteria are presented in Textbox 1. Participants were chosen without consideration of whether they had previously interacted with the COVID Alert app.

**Table:** Textbox 1

**Inclusion criteria**
18 years of age or older
Able to read and speak in French
Has a good Internet connection, given the virtual format of meeting through a videoconferencing platform
**Exclusion criteria**
Holds one of the following positions: elected at the federal, provincial, municipal or school levels
Is an employee, representative or shareholder of a company involved in technology development, or is a family member of a person holding one of these positions

Involvement of the patients and citizens included taking part in five two-hour meetings which, given the COVID-19 pandemic, were held online. The first one was an information meeting, the second and third ones were co-construction sessions of the questionnaire, and the last two meetings were dedicated to the discussion of survey results and formulating recommendations.

Before the first meeting, the participants had access to an online shared file that contained the program of the meeting, popularized texts on exposure notification applications, and four short video clips produced by the research team to introduce the project and discuss technical, ethical, and legal aspects of such technologies. During meeting #1, we first discussed the rules of engagement to ensure a safe and respectful environment for participation by all committee members. We asked that participants remain open to each other's interventions, be concise in their comments to allow sufficient time for other participants, and use the ‘raise hands’ feature in Zoom. They were invited to intervene when they wanted and felt they had something to add to the conversation. Then, we discussed the project objectives and what needed to be done before starting to co-construct the questionnaire. Moreover, the participants were invited to write down ideas in a shared online document about concepts to include in the survey. Finally, the researchers suggested the idea of enhancing communication with participants and sharing relevant news or articles regarding digital contact tracing and exposure notification apps by creating a workspace on an online messaging software.

#### Findings

A total of 25 applications were received, all of which were evaluated by the research team. Sixteen people were selected for interviews guided by the inclusion criteria, of which 12 were selected to take part in the advisory committee. Regarding the criteria for representativity and diversity, they formed different age groups and were from seven geographic regions across the province of Québec. Half of the participants were patients (having medical conditions that increase the risk of complications from COVID-19), and seven were men. Two participants were less than 30 years old, two were between 40 and 50 years old, three were between 50 and 65 years old, and four were more than 65 years old. Two of the participants were living with a disability, two identified as a visible minority, and one worked with homeless people and Indigenous communities. At the end of the project, each participant with a minimum attendance of three meetings received $500 for their participation.

During meeting #1, participants suggested creating a shared document in which everyone could write down the questions regarding the survey methodology, that the research assistant could then send to the polling firm in charge of data collection. Shortly after, 17 questions were included in this document. Moreover, the participants raised the important point of increasing the representativeness of survey respondents, since they would come from the polling firm’s panel. They suggested conducting focus groups with marginalized groups or people working with vulnerable groups to supplement the survey results. Thus, a shared document was created to jointly make a list of organizations and partners that we could contact and with whom we could collaborate. After a few weeks, six contacts were proposed. For the concepts to be effectively integrated into the questionnaire, 15 ideas were added by participants in the shared document. Finally, a workspace was also created on an online messaging software to enhance communication within the advisory committee, but only seven out of twelve participants joined the group, and only two posts were made by participants, the rest being from the research assistant and principal investigators.

### Step 2: Co-construction of the Questionnaire (February to May 2021)

#### Methods

Two meetings were dedicated to the co-construction of the questionnaire, in which representatives of SOM, an independent research firm mandated to program the survey, took part. In the first one (meeting #2), to formulate the questions, the advisory committee was divided into three breakout rooms, to discuss three main themes regrouping the 15 ideas proposed after meeting #1: (1) the motivations to use such tools, (2) the concerns to use or to download the app, and (3) the conditions for social acceptability. Each room had a host (M.P.P., E.O., or S.B.) who moderated and summarized the discussions. The hosts changed rooms after 10 min, and the subgroups would continue to formulate questions within the theme presented by the new host. Once each of the three groups had participated in the three themes, all the participants returned to the main room and reviewed the questions that stemmed from this process.

After meeting #2, a preliminary version of the questionnaire combining the questions of the three themes was created, which was revised by the research team. Meeting #3 aimed to prioritize and rephrase questions via the same process as in meeting #2. After collating the questions from each theme in meeting #3, in the following weeks, we had four 30-min work sessions where members of the advisory committee and the research team selected the final items prior to questionnaire programming.

For the pretest, the link to the questionnaire was then sent to the advisory committee, the research team, and within a patient partner advisory council (Conseil des citoyens partenaires en santé (COCIPS) at the CEPPP. In addition, all members of the research were invited to two additional optional drop-in sessions to review the nearly final version of the questionnaire. Comments and suggestions could be sent to the research team. The survey was distributed from May 27 to June 28, 2021, by SOM.

#### Findings

In meeting #2, a total of 84 questions were generated in the three 10-min brainstorming sessions. In meeting #3, the questionnaire was reduced to 44 questions. After the drop-in sessions, the final version of questionnaire contained 36 items, excluding 8 socio-demographic questions. The specific concepts integrated into the final version of the questionnaire included (1) the use of a smartphone and the COVID Alert app, including access to a smartphone, experience with the COVID Alert app, and influences on the decision whether or not to download the app, (2) knowledge of the respondent regarding the COVID Alert app, and (3) perspectives about the COVID-19 pandemic and digital contact tracing and exposure notification apps, including motivations and barriers on the decision whether or not to download the app, personal risk perceived regarding the SARS-CoV-2 virus, and citizens’ involvement in the development of the app.

Regarding the survey, a total of 2,249 invitations were sent by email, of which 959 adults agreed to participate. In total, 859 questionnaires were filled via the web survey and 100 via the telephone survey. The results are presented in Osmanlliu et al. [23].

### Step 3: Discussion of the survey results (August to November 2021)

#### Methods

After the survey was carried out, the results were presented in meeting #4 to allow the participants to react to the findings, give their explanations to interpret them, and explore how the analyses could be deepened. Following their recommendations, a statistician conducted data analysis and statistical modeling.

During the final meeting (#5), to which all members of the research team were invited, the final results were presented. The goal of this session was threefold: (1) to assess the different interpretations of the results, (2) to prioritize the elements to be included in an article presenting the survey’s results [23], and (3) to establish recommendations to ensure sustainable and socially acceptable use of such an application in the future. To facilitate the discussion, we used the same method of breakout rooms, one for each goal.

#### Findings

During meeting #4, the participants shared their impressions. For instance, they highlighted that respondents were more likely to endorse mandatory app enforcement when the risk perception of a public health crisis was greater. Also, they made five suggestions on how to further analyze data, such as cross-referencing the data of the perceived level of knowledge of digital contact tracing and exposure notification apps and concerns of the populations.

In meeting #5, they discussed 25 ideas for recommendations to help increase the app uptake in the population, based on their own interpretations of the survey results. For example, they suggested developing media campaigns tailored to the concerns and needs of specific age groups, particularly teenagers. They also recommended that other strategies for contact tracing for people without access to smartphones be implemented and that functions in design for more active use of the app by users be added. With respect to the DIGICIT project, they suggested writing this article on the co-construction process, as they recognized the academic value of the co-construction methodology.

### Step 4: Assessment of patients’ and citizens’ experience (March and September 2021)

#### Methods

We sent the participants a small survey after meeting #2 to evaluate the method and assess their experience in the advisory committee. Three open-ended questions were asked: (1) what they most appreciated about their participation in the first two meetings, (2) what they did not like about the first two meetings and what improvements could be made for the next ones, and (3) what facilitated everyone's participation considering the virtual format. All responses were anonymous.

In addition, we invited participants by email to partake in individual interviews in a virtual format to highlight their experiences. The interviews were held after meeting #4. The questions focused on their motivations to participate in the advisory committee and their expectations, their satisfaction after having participated, and how the research partnership process could be improved in a future project. The interviews were recorded with the participants’ consent and transcribed in their entirety for analysis.

Ultimately, at the end of each meeting, we asked the participants to share their overall appreciation of their experience participating in the advisory committee. We also asked about how future meetings could be improved.

#### Findings

##### The survey

Seven participants filled out the survey. What they most enjoyed in the meetings were the breakout rooms, as they facilitated exchanges between participants. They found that smaller groups enabled more equal speaking time, thus everyone had the chance to express themselves. They also sensed that all opinions matter and that everyone can share and receive respect from others. Other elements cited included the topic of the project and the ease of communicating in virtual format. Finally, one person stated that they particularly enjoyed their "inclusion early in the process to solicit our ideas for this survey."

When asked about what they least enjoyed, they responded that some participants’ interventions were sometimes too long in the introduction meeting, that some people talked more than others, and some interventions were not always in tune with the topic of the moment. However, one participant mentioned that speaking time allocation was improved in the first co-construction meeting. Moreover, they thought that the project’s objectives were not always clear, as well as the method used to achieve them. Some also felt that the time allocated for the survey development was too short and "even a little rushed," while the time spent in the main room sharing the questions generated in the brainstorming sessions was a little too long. Finally, one participant mentioned that they wished to see more interactions in the online messaging software between meetings.

Finally, we asked participants about how we could improve their experience with the virtual format. Many said that everything worked well on that account, that it was already dynamic, and that the means used were adequate (such as having access to the meeting’s agenda with a schedule, being in subgroups which gives more people an opportunity to express themselves, and the "raising hands" feature, although some suggested that it be used more systematically). However, one participant suggested "asking people to rename themselves so that the identity of each was clear, and directly engage people who have not yet spoken." Other ideas include opening the meeting half an hour before the start of the meeting for people who wish to socialize and having a maximum time per intervention.

##### The interviews

Regarding the individual interviews with participants, three agreed to be interviewed to highlight their experience. The interviews lasted between 35 and 50 min. A summary of the results can be found in Table 1.

**Table 1:** Participants’ main responses to interview questions

Why they participated	What they enjoyed	What could be improved
Pandemic affects them as patients	The topic	Clearer tasks and objectives in meetings
Interest in getting involved	The brainstorming subgroups	Knowing the date of the meetings more in advance
To better understand other perspectives	The chance to express themselves	More balanced speaking time between participants
To make sure the interests of users and vulnerable groups are met	The collective intelligence	More time for brainstorming sessions
Curiosity about the co-construction process	Their inclusion early in the research process The opportunity to make an impact	More time for discussions More diversity in the committee

##### Overall appreciation

Generally, they reported having a great experience. After meeting #2, they said that the meetings were stimulating and that it was promising for the rest of the project: "It’s a very pleasant meeting, it’s always more stimulating to participate when it's interactive, and I just can't wait to get started." Some also stated that they formed a nice group of people with diverse opinions, which enabled them to go further into the discussions. One person said that they were surprised by the energy of the moderation and activities and that they "didn't expect it to be so open for discussion."

With respect to the virtual format, they said that it can generally be less interesting than in-person gatherings, but they have found that these ones were enjoyable. Also, for participants living with a disability, a virtual format made it easier for them to participate in. Some also found that separating the group into smaller breakout rooms for the brainstorming sessions was efficient and productive. Indeed, they reported that there was "a wealth of material that emerged in little time." They also said that the activity was well structured and that it allowed them to discuss all three themes.

In regard to the improvement of the meetings, one participant suggested "having a discussion on the general vision of the questionnaire before going into the sub-themes." Others also suggested that a clearer definition of their role prior to the activities would have been useful, as some felt that "there was no feeling of being able to participate fully because we did not know what was required." Regarding the interpretation of survey results, many participants identified the need to obtain a synthetic version of study findings at least one week prior to the group meeting, in addition to a summary presentation during that meeting.

Two members of the advisory committee, who also co-authored this paper, further reflected on their own participation in the co-construction process. One said they were struck that even if no one around the table had expertise in the design of such an application, almost all the issues, the important and the less important ones, were raised by citizens and patients. The other suggested that this could partly be explained by patients and citizens having different expertise than the research team. This diversity of expertise led to the selection of themes chosen meeting #1 that necessarily reflected the patients’ and citizens’ own experience and made them think about how the questions of the questionnaire would be received by people like them. Finally, both were convinced that the creators of the COVID Alert app had a lot to gain from involving patients and citizens in their approach, for a minimal budget and without having to extend the deadline.

## Discussion

Citizens and patients are rarely consulted and included in digital health research or in the development process of techno-scientific tools, such as digital contact tracing and exposure notification apps [24]. The DIGICIT project is one of the rare studies to practice partnership [14] and to engage patients and citizens in the questionnaire co-construction, interpretation of the survey results, and knowledge translation efforts. Having patients and citizens actively participating in this research constitutes the main methodological strength of the DIGICIT project, and had a positive impact on the study. Indeed, their discussion on the representativeness of the survey results and their suggestion to conduct focus groups led to the addition of a new step in the research protocol. Diverse perspectives are essential to responsible digital health research, given their potential to exacerbate existing social inequities. We sought to enrich perspectives at the survey development level, by establishing diversity criteria that informed the selection of participants; at the survey distribution level, through a sampling strategy that promoted a provincially representative sample across various sociodemographic factors; and thanks to the advisory committee, through focus groups among groups in situations of vulnerability, as they are usually under-represented in traditional surveys. This shows how their perspectives contributed to a key dimension of the DIGICIT project that might have otherwise been overlooked [16]. Moreover, they suggested writing an article on the co-construction process, because they found it was an interesting method where a lot of material was generated in a short period of time and thought that it should be known to the scientific community. As a result, their suggestion led to this paper being drafted. This is a great example of Torfing et al. [11] recommendations to use different kinds of knowledge, resources, competencies, and ideas to foster more efficient and effective solutions through collaborative efforts.

The participants' evaluation of their own implications in the DIGICIT project highlights that the meetings were highly productive. Indeed, there was a substantial number of survey questions generated in a short period of time and the topics were decided by mutual agreement. Ideas were retained by group consensus, through discussion in large and small-group settings. The rich creativity was of great use in interpreting the survey results and planning knowledge translation initiatives. More pre-meeting preparation could have improved the efficiency of meetings. For example, participants highlighted the need to share survey results sufficiently in advance for review by the advisory committee members.

Our results support the six actions proposed by Wilson et al. [13] to achieve a positive impact of PPI in research, and we add concrete strategies applied and learned in the co-construction process for best practices. First, it is suggested that lay representatives must have a strong connection with the target study population (action 3). This was achieved by integrating 12 non-professional people who participated in the co-construction advisory committee of the survey questionnaire of the DIGICIT project. However, to create a stronger connection, we find it important to involve patients and citizens from the start of the research project. This is something that was appreciated by some members of the advisory committee. It was achieved by having a patient (S.B.) as a principal researcher.

Other actions proposed by Wilson et al. [13] include that the researchers and participants have a shared understanding of the moral and methodological purpose of PPI (action 1) and that the whole research team must be positive about PPI input and fully engaged with it (action 4). In the DIGICIT project, both actions were achieved by having the researchers work closely with the advisory committee, which enabled its members to participate in the common definition of the research methodology. However, there was a tension between the desire not to define the specific objectives before the first meeting to maintain an openness to suggestions, and the need to clarify the objectives of the project and the role of the citizens and patients. This constituted a learning point for us, which can, however, be improved by a continuous and dynamic evaluation of the process during the project. Concretely, we find it important to clearly explain the objectives of the project to participants and the method used to achieve them, as well as the tasks during brainstorming sessions. Above all, enough time must be allocated to the meetings to discuss and modify them as needed.

The second action proposed by Wilson et al. [13] is to have a key individual coordinating PPI. This was achieved in the DIGICIT project by having a research assistant who coordinated all meetings and ensured the follow-up of the projects with all the members of the team, including participants. A rigorous preparation and organization of the meetings can further support the success of this process. In this project, it included setting the dates of meetings in advance, sharing an agenda, and establishing clear rules for speaking in those meetings (duration, raising your hand, respect for others). Additionally, a research team member should be assigned to moderate the meetings to ensure that participants do not exceed the allotted speaking time and encourage participation among people who have not yet had the opportunity to speak.

Moreover, it is suggested by Wilson et al. [13] that efforts to develop relationships must be established and maintained over time (action 5). Since, in the DIGICIT project, the meetings took place over more than six months, we had to create strategies to reinforce participation over time, such as surveys and interviews. Additionally, in an attempt to encourage discussion outside of meetings, a workspace on an online messaging software was created for the participants, but only a few people were more actively involved. To reach the entire group, messages and updates on the project were instead sent by email where the response rate was higher.

Finally, the last action that is suggested by Wilson et al. [13] is to evaluate PPI proactively and systematically. Of course, it is crucial to have an ongoing assessment of participants’ experience and to understand how the research team can improve at integrating them. This was achieved by asking at the end of each meeting for their impressions about the activities and their feeling about their integration in the project, by sending them a small survey after meeting #2 to give them the opportunity to comment anonymously on the ways in which the meetings could be improved, and by giving them the opportunity to participate in an interview after meeting #4 to further discuss their thoughts on the co-construction process.

### Limitations and strengths

We consider having patients and citizens actively participating in this research as our principal strength, as well as having an advisory committee composed of people of diverse profiles to include a range of perspectives, as this methodology can be used in various clinical contexts and different healthcare settings. Indeed, this methodology underlines the role of partnership in the process of health research, in addition to suggesting its usefulness in the development process of digital health apps. However, people with greater limitations could have been incapable of participating in the project, thus creating a selection bias. To palliate the need to include a wider range of perspectives, we tried to reach people from communities that are historically excluded from research. However, in practice, we had difficulty reaching Indigenous people, people with limited literacy, and homeless people. Some of these difficulties can be attributable to the context of the pandemic, where some organizations were preoccupied with pressing issues affecting their members and lacked the resources to participate. On the other hand, the study topic was perceived as being too disconnected from the people who do not have access to this type of technology. It seems that these organizations have mainly retained the technological aspect and less that of concern for systematic exclusion, in part due to gaps in digital literacy and access. In the future, a more open and adapted form of communication, highlighting the direct impacts of the research theme on a particular group rather than on the specific technology, may be a more effective way of connecting with community organizations. Explicitly valuing and seeking the experience of marginalized groups may also help. By discussing with the managers of an organization supporting individuals with a limited level of literacy, we found that a major barrier to their participation in a discussion session was the perception that their contribution would be limited. However, these perspectives are essential to better grasp the understanding, fears, expectations and accessibility needs of a group that may be otherwise excluded from this type of innovation. Fortunately, we were able to meet with a group of immigrant women, offering a diversity of perspectives on the relevance of such an application in their context.

## Conclusion

In conclusion, the DIGICIT project underscores the importance of involving patients and citizens early in the process so that the research reflects the concerns of key stakeholders that might otherwise have been overlooked. Patients and citizens involved in the advisory committee improved the quality of the process of creating the questionnaire by adding dimensions they considered essential. We showed that co-construction in digital health research can be quick and efficient in bringing out a substantial amount of information on different issues through collective intelligence. Patients and citizens also had a great impact on the study by adding a complete pan-step in the research protocol to carry out focus groups with marginalized populations to complete the survey data and make it more representative. Clear communication of the project objectives, good organization in meetings, and continuous evaluation from the participants allow best practices for patient involvement in digital health research.

## Supplementary Information

**Additional file 1**. GRIPP2-Long Form Checklist for the Reporting of Patient Engagement in Research.

## Data Availability

Data sharing is not applicable to this article as no datasets were generated or analyzed during this study.
